# Cloning, Expression, and Characterization of a Metalloprotease from Thermophilic Bacterium *Streptomyces thermovulgaris*

**DOI:** 10.3390/biology13080619

**Published:** 2024-08-15

**Authors:** Amna Mushtaq, Sibtain Ahmed, Tahir Mehmood, Jorge Cruz-Reyes, Amer Jamil, Shafaq Nawaz

**Affiliations:** 1 Department of Medical Laboratory, Times Institute, Multan 60000, Pakistan; amnamushtaq.pak@gmail.com; 2 Department of Biochemistry, Bahauddin Zakariya University, Multan 60800, Pakistan; 3 Institute of Microbiology and Molecular Genetics (IMMG), University of the Punjab, Lahore 54590, Pakistan; tahir.mmg@pu.edu.pk; 4 Department of Biochemistry and Biophysics, Texas A&M University, College Station, TX 77843, USA; jorge.cruz-reyes@ag.tamu.edu; 5 Department of Biochemistry, University of Agriculture, Faisalabad 38040, Pakistan; 6 Department of Zoology, Government College Women University, Sialkot 51310, Pakistan; shafaqnawaz605@gmail.com

**Keywords:** neutral protease B, thermophile, *Streptomyces thermovulgaris*, proteolytic enzyme, cloning, expression and characterization

## Abstract

**Simple Summary:**

Proteases are vital enzymes that break down proteins into smaller units, peptides, or amino acids, playing a role in several biological processes. Due to various industrial applications, including detergents, laundry, leather, and pharmaceutical industries, the production of thermostable proteases is of great concern. Thermophilic bacterium *Streptomyces thermovulgaris* produced the metalloprotease in a bacterial expression system. Overexpression was achieved using an inducer in 4 h, yielding a soluble protease. Protein was purified by precipitation and an affinity chromatography system and was stable. It retained 80% activity at a wide range of pH and temperature with a half-life of 4 h, which is highly efficient for its use in various industries.

**Abstract:**

Proteases hydrolyze proteins and reduce them to smaller peptides or amino acids. Besides many biological processes, proteases play a crucial in different industrial applications. A 792 bp protease gene (*nprB*) from the thermophilic bacterium *Streptomyces thermovulgaris* was cloned and expressed in *E. coli* BL21 using pET 50b (+). Optimal recombinant protease expression was observed at 1 mM IPTG, 37 °C for 4 h. The resulting protease was observed in soluble form. The molecular mass estimated by SDS-PAGE and Western blot analysis of the protease (NprB) fused with His and Nus tag is ~70 KDa. The protease protein was purified by Ammonium sulfate precipitation and immobilized metal ion affinity chromatography. The optimum pH and temperature for protease activity using casein as substrate were 7.2 and 70 °C, respectively. The mature protease was active and retained 80% of its activity in a broad spectrum of pH 6–8 after 4 h of incubation. Also, the half-life of the protease at 70 °C was 4 h. EDTA (5 mM) completely inhibited the enzyme, proving the isolated protease was a metalloprotease. NprB activity was enhanced in the presence of Zn^2+^, Mn^2+^, Fe^2+^ and Ca^2+^, while Hg^2+^ and Ni^2+^ decreased its activity. Exposure to organic solvents did not affect the protease activity. The recombinant protease was stable in the presence of 10% organic solvents and surfactants. Further characterization showed that zinc-metalloprotease is promising for the detergent, laundry, leather, and pharmaceutical industries.

## 1. Introduction 

Many microbial genera from bacteria and fungi have been found to produce industrially essential enzymes [1,2,3]. Proteases are among the largest group of enzymes with crucial industrial applications. Proteases are synthesized by various microorganisms, including bacteria, fungi, yeast, molds, animals, and plants [4]. Proteolytic enzymes are ubiquitous in occurrence and are essential for biological processes such as cell growth, cell signaling, proliferation, differentiation, and immune response [5,6]. Proteases are the most important class of enzymes, constituting approximately 60–65% of the total industrial enzyme market. Globally, protease enzymes are estimated to cost USD 3 billion [7,8]. The proteases, especially neutral proteases, have many applications around the world in various industries and make up the most significant proportion of the industrial enzymes [9], detergents [10], food [11], feed [12], pharmaceutical [13], textile, leather, and silk industries because of their distinct benefits including low potential level, high yield and mild catalysis process [14,15]. In the food industries, neutral proteases are generally used for de-bittering beer brewing and soy sauce [16].

Metalloproteases are the most diverse of all proteases and are generally referred to as camelysin [17]. They require a divalent metal ion for their activity and are inhibited by chelating agents such as EDTA [18]. Microbial metalloproteases show high proteolytic activity and industrial application and are a source of green additives. Metalloproteases contain Zn^2+^, Ni ^2+^, Co^2+^, Cu^2+^, and other metal ions in the centers of their active sites as functionally essential parts to catalyze the hydrolysis of peptide bonds. In the catalytic process, these metal ions activate water molecules for nucleophilic attack on the carbonyl group of the peptide bond, thus facilitating substrate hydrolysis [19].

Neutral protease B (NprB) is one of the most thermostable metalloprotease enzymes produced by *Streptomyces thermovulgaris*, a thermophilic bacterium that optimally grows at pH 7.0 and 55 °C. This species produces multiple protease enzymes when it grows on rape meal [20].

In this study, we describe the cloning of a thermophilic neutral protease (*nprB*) gene from *S. thermovulgaris*, its expression in *E. coli*, and the purification and characterization of the recombinant protease. 

## 2. Materials and Methods

### 2.1. Strains, Media, and Culture Growth 

Bennett’s agar medium was used to maintain *Stretomyces thermovulgaris* NBRC 12383. The bacterial culture was grown aerobically at pH 7.3, 45 °C for 24 h. The strain was further grown on malt/yeast extract medium [21] for DNA isolation. 

### 2.2. Cloning and Expression of nprB Gene

The PCR was run in a thermocycler (BioRad) for 30 cycles: denaturation for 30 s at 94 °C, annealing for 1 min at 56 °C, and extension for 1 min at 72 °C using 3′-GATCGGCTGGCCAGAATAG-5′ and 3′-AGGCGAGATCATATTCACCG-5′ as forward and reverse primers, respectively. The primers were designed using *Bacillus* subtilis; accession no. AJ973636.1, which shows a 96% similarity index (BLASTn). The PCR-amplified protease gene was sequenced.

Using a standard protocol, the purified protease gene was cloned into the pTZ57R/T vector using a Thermo Scientific (Waltham, MA, USA) InsTAclone PCR cloning kit (catalog number K1231). The protease (*nprB*) gene was excised with *Kpnl* and *SalI* restriction enzymes and ligated into a pET-50b (+) vector for expression. The expression of the protease gene was observed at varying concentrations of IPTG viz 1.0 mM to 10 mM. The cells were harvested after different incubation periods (i.e., 2, 4, 6, 8, 10, and 12 h) and sonicated (15 s pulse and 15 intervals, 20 cycles using Sonica–Vibra cell, Newtown, CT, USA) using phosphate buffer (50 mM; pH 7.4) containing 20 mM imidazole. The extract was centrifuged at 11,500× *g* for 40 min at 4 °C, and the supernatant was tested for protease assay [22].

### 2.3. Purification of Recombinant Thermophilic Neutral Protease 

The crude extracellular enzyme extract from overnight culture of *E. coli* was precipitated at 80% ammonium sulfate saturation (*w*/*v*). Along the purification pathway, UV–visible spectroscopy, SDS-PAGE, and Western blot measurements were carried out to determine the quality and efficiency of the purification and expression. 

The protein was further purified by FPLC (GE Healthcare (Chicago, IL, USA), AKTA, Amersham Pharmacia Biotech, Amersham, UK). The recombinant NprB was purified by affinity chromatography using a Ni-bound Hi-Trap FPLC column. The purified protease was analyzed by SDS-PAGE. The two buffers were used for the purification. Potassium phosphate (50 mM, pH 7.5, 5 mM imidazole, 500 mM NaCl) was used as buffer A, and potassium phosphate (50 mM, 500 mM NaCl, 500 mM imidazole) was used as buffer B. The purified protein was subjected to SDS-PAGE [23]. The cell pellets were resuspended in a sample buffer of 1X SDS-PAGE (Invitrogen, Carlsbad, CA, USA), boiled for 5 min, loaded 20 µL of the sample on 12% of the gel, and electrophoresed for 1 h at 200 V on Mini-Protean II electrophoresis cell (Bio-Rad, Hercules, CA, USA). The gel was stained with Coomassie blue R-250 for 30 min and destained with 40% methanol and 10% acetic acid with several solution changes [24].

### 2.4. Western Blot Analysis of Thermophilic Neutral Protease

After separating proteins by SDS-PAGE, the protein was transferred to the nitrocellulose membrane on a Mini Trans-Blot electrophoretic transfer cell (Bio-Rad) at 30 V/100 mA overnight. Protein transfer and blotting were done using a standard protocol of Western blotting [25]. Rabbit–anticamelysin (1:5000) and anti-rabbit IgG- alkaline phosphatase (Sigma (Kawasaki City, Japan)) were used as primary and secondary antibodies [26].

### 2.5. Protease Activity Assay

The proteolytic activity of the protease enzyme was determined using 0.5% casein in potassium phosphate (50 mM) of pH 7.2 as a substrate and incubated with enzyme solution (100 µL) for 10 min at 70 °C. The reaction was stopped with 10% TCA and centrifuged at 12,000 rpm for 10 min. The absorbance was noted at 660 and 280 nm. One unit of proteolytic protease activity was defined as the amount of enzyme required to release 1 μg of tyrosine per minute under the experimental conditions [27].

### 2.6. Characterization of Thermophilic Neutral Protease

#### 2.6.1. Determination of Optimum pH and pH Stability

The effect of pH on the activity of protease was determined in potassium phosphate buffer (50 mM) at optimum temperature with different pH values (i.e., 5–10) at optimum temperature following the enzyme assays [10].

#### 2.6.2. Determination of Optimum Temperature and Thermal Stability

The optimum temperature was determined by measuring the enzyme activity at 30–100 °C as described in the enzyme assay. Enzyme stability was determined by incubating the enzyme reaction for 30 to 240 min with an increase in a time interval of 10 min at optimum temperature in 50 mM potassium phosphate buffer of pH 7.2 [27].

#### 2.6.3. Enzyme Substrate Specificity and Kinetic Parameters (*K_m_* and *V_max_* Determination)

The enzyme specificity was estimated by measuring the enzyme activity with various substrates (e.g., BSA, azocasein, and casein). Different substrates of 0.5% concentrations were prepared in a 50 mM potassium phosphate buffer of pH 7.2 under standard substrate conditions. The absorbance changes were recorded spectrophotometrically at 280 and 660 nm to obtain the residual activity [28].

The kinetic parameters Michaelis–Menten constant (*K_m_*) and the maximum rate (*V_m_*) of the enzyme activity related to the recombinant enzyme were calculated through double reciprocal Lineweaver–Burk plot equation of protease activity towards casein as substrate.

Casein, azocasein and BSA were used as a substrate. A kinetic analysis of NprB protease enzyme reaction was carried out under optimal reaction conditions. The kinetic parameters Km and Vmax were calculated using the Lineweaver–Burk plot method. In comparison, Kcat = Vmax/[E], where [E] represents enzyme concentration [29].

#### 2.6.4. Effect of Various Metals Ions and Organic Solvents 

The effect of metal ions viz Mg^2+^, Fe^3+^, Cu^2+^, Zn^2+^, Ca^2+,^ and Mn^2+^ on protease activity was determined in the presence of metal ions (10 mM) following the protease assays [10]. The effects of solvents methanol, ethanol, n-butanol, benzene, hexane, toluene, chloroform, and dimethyl sulfoxide (DMSO) on protease activity were measured by incubating the enzyme with 10% of each solvent. The protease activity without solvent served as the control, which was considered 100% activity [10].

#### 2.6.5. Effect of Detergents and Inhibitors on Protease Activity

The effects of various surfactants, including SDS, Tween-80, Tween-40, Trion X-100, H_2_O_2_, metal ion chelators viz ethylenediaminetetraacetic acid (EDTA), iodoacetamide, dithiothreitol (DTT) and iodoacetamide, and enzyme inhibitors viz phenylmethylsulfonyl fluoride (PMSF), ethylenediaminetetraacetic acid (EDTA), dithiothreitol (DTT), iodoacetamide, pepstatin A, cetrimonium bromide (CTAB) and dimethyl sulfoxide (DMSO) were studied. The concentration of all the agents used in the assay mixture was 1 mM [30].

## 3. Results 

In this study, an extracellular thermophilic neutral protease from a thermophilic bacterium, *S. thermovulgaris*, was cloned, expressed, purified, and characterized with promising industrial applications.

### 3.1. Cloning and Sequence Analysis of Neutral Protease B (nprB)

A potent protease-producing thermophilic bacterium *Streptomyces thermovulgaris* NBRC 12,383 was investigated due to its great potential for protease production [20]. The DNA was isolated from *S. thermovulgaris,* followed by PCR amplification of a 792 bp open reading frame (ORF), which was confirmed as a protease gene (*nprB*) by sequence analysis. The gene sequence was deposited in GenBank, and accession No. KX879552 was allotted. Different online tools and software were used to characterize neutral protease B’s gene and protein sequence [31]. The *nprB* gene was first ligated into a cloning vector pTZ57R/T and then transferred into *E. coli* DH5α for further analysis. 

### 3.2. Expression and Purification of Recombinant Neutral Protease B

**Figure 1:**
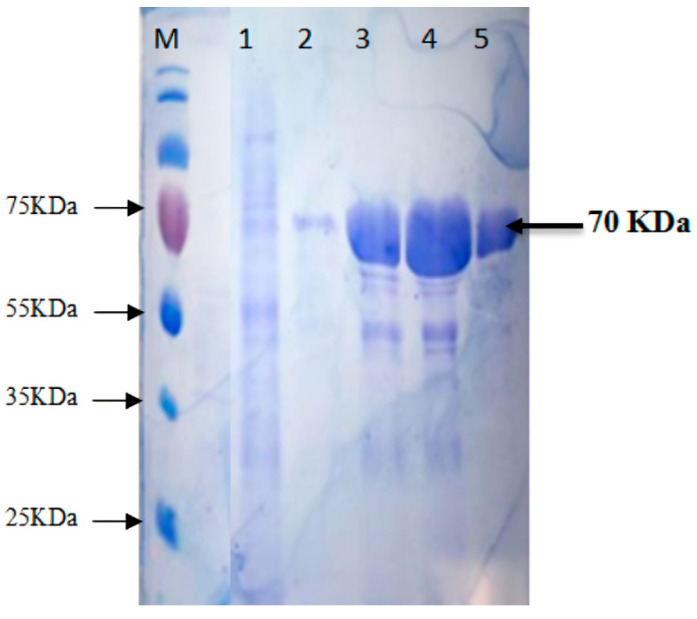
SDS-PAGE for the protein expression in *E. coli* BL21 (Rosetta-gami pLysS) DE3 harboring pET-50b (+) construct (Coomassie blue staining). Lane M is a protein molecular weight (MW) ladder (Precision Plus protein, Bio-RAD), Lane 1 = uninduced (nprB) pET50b (+) construct. Lane 3–5 = IPTG-induced (nprB) pET50b (+) construct. Lane 2 = Ni-coated Hi-trap FPLC-purified protease protein (~20 kDa) fused with Nus + His-tag (~50 kDa), making a total ~70 kDa.

The recombinant protease gene from pTZ57R/T was sub-cloned in an expression vector pET-50b (+). The gene was successfully expressed as a soluble protein along with the N-terminal Nus tag and His-Tag in *E. coli* BL21 (Rosetta-gami pLysS, Merck KGaA, Darmstadt, Germany) DE3 Cells. The maximum expression of the protein using 1 mM IPTG induction was identified after 4 hrs incubation time at 37 °C. The SDS-PAGE revealed the fused protein’s molecular mass to be ~70 kDa; protease of ~20 kDa fused with Nus/His tag of ~50 kDa (Figure 1).

**Figure 2:**
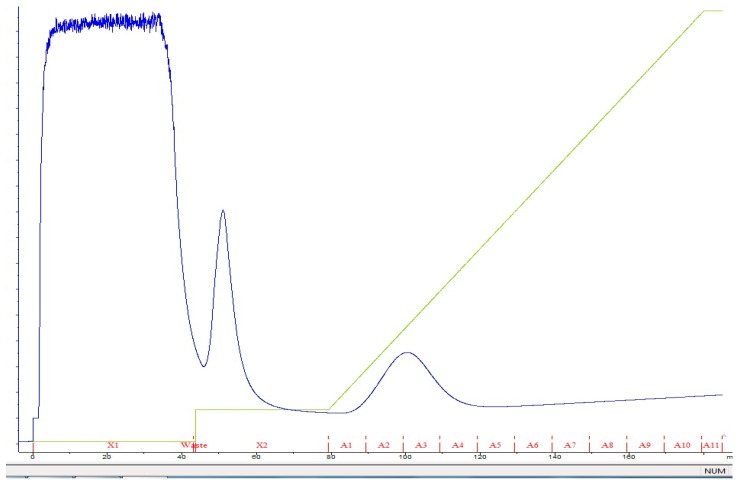
FPLC chromatogram of recombinant neutral protease B (NprB) cloned in pET-50b (+) and expressed in *E. coli* BL21 (Rosetta–gami pLysS) DE3. Fraction elution A2 and A3 showed positive results.

**Figure 3:**
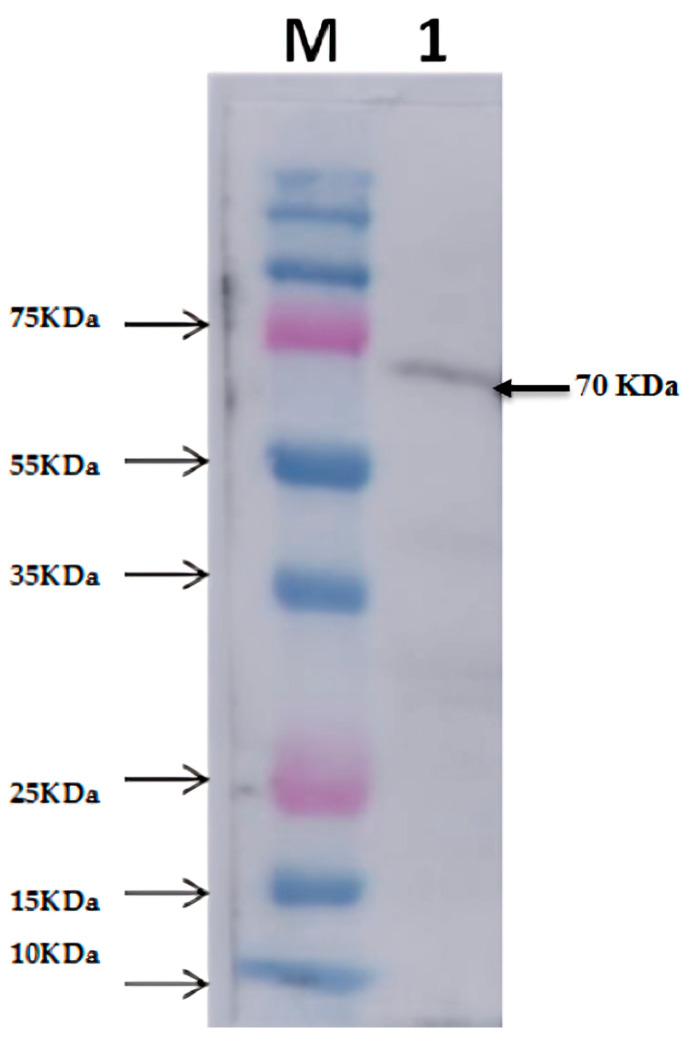
Western blot analysis for protease expression extracted from the *E. coli* BL21 (Rosetta–gami pLysS) DE3 harboring recombinant (nprB) pET50-b (+) at 1 mM IPTG induction and run on 12% SDS-PAGE. Lane M protein molecular weight (MW) ladder (Thermo Scientific page Ruler). IPTG Lanes 1 = Transferred protein on nitrocellulose using rabbit-anticamelysin (1:5000) and anti-rabbit IgG- alkaline phosphatase (Sigma) (protease protein (~20 kDa) fused with His-tag and Nus-tag protein (~50 kDa) making a total ~70 kDa).

The protease protein from *S. thermovulgaris* was purified using a three-step procedure. The sonicated culture filtrate was concentrated in ammonium sulfate at 20 to 80% of saturation at 4 °C. After this step, approximately 80% of the initial protease activity was concentrated (Table 1). The crude extract after ammonium sulfate precipitation exhibited multiple protein bands on SDS-PAGE (Figure 1). The precipitated pellet was dissolved in a minimum volume of 50 mM potassium phosphate buffer (pH 7) and the protein was further purified by affinity chromatography using Ni bound Hi-Trap FPLC column (Figure 2). The purified protease after Western blot analysis revealed a molecular weight of fused protein ~70 kDa shown in Figure 3. 

### 3.3. Characterization of Recombinant Neutral Protease B

#### 3.3.1. pH, Temperature Optima, and Stability

**Figure 4:**
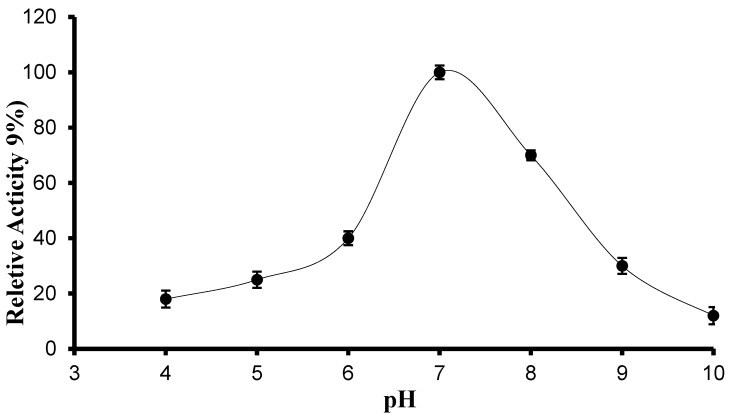
The effect of pH on the recombinant neutral protease B protein relative activity. The temperature used to investigate the pH effect was 70 °C. Error bars represent the standard deviations of three measurements.

The effect of pH on the recombinant protease activity was estimated by measuring the activity in different buffers with different pH values ranging from 5.0 to 10. A bell-shaped curve with the highest activity at pH 7.0 was observed (Figure 4). The protease enzyme exhibited lower enzyme activity at acidic pH than at neutral or alkaline pH. The enzyme lost 25% of its activity at pH 5 and 15% of its activity at pH 9. It was revealed by comparing protease activity at various pH values that the enzyme is nearly stable at different conditions but shows high activity at neutral and alkaline pH values. 

**Figure 5:**
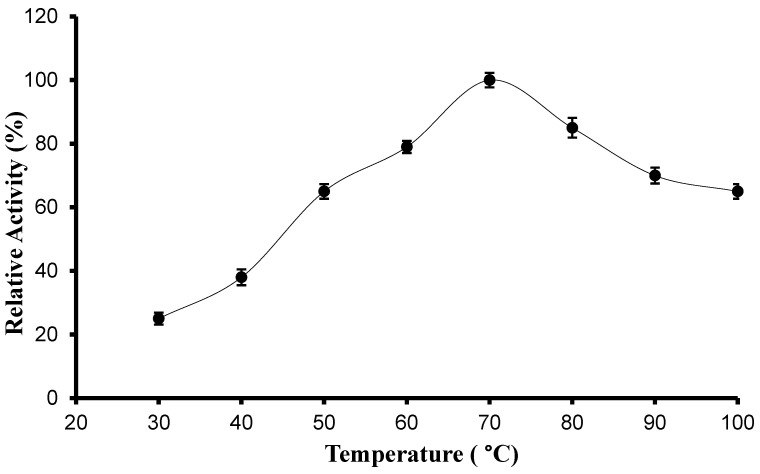
The effect of temperature on the activity of neutral protease B. Error bars represent the standard deviations of three measurements.

The effect of temperature on protease activity was estimated by measuring the enzyme activity at different temperatures in the range of 25 °C–100 °C. The bell-shaped curve shows the temperature effect with the highest protease activity at 70 °C (Figure 5). When the protease activity was compared at different temperatures, it was revealed that the neutral protease B is stable at the given conditions as it shows >75% of its activity at all these temperatures. 

The stability of the recombinant protease was investigated by incubating the enzyme reaction at different temperatures ranging from 50 to 95 °C for 6 h. The enzyme retained more than 95% of its activity at 60 °C after incubation of one hour and retained approximately 30% of its activity after the incubation of six hours. At the temperature of 90 °C, the enzyme retained more than 80% of its activity after incubating one hour and 20% of its activity after the incubation of 6 h. These results indicated that recombinant neutral protease B isolated and purified from the thermophilic bacterium *S. thermovulgaris* is stable at a temperature range of 60–95 °C during the incubation time of six hours with no significant loss in the enzyme activity. 

#### 3.3.2. Substrate Specificity and Kinetic Properties 

Substrate specificity of the neutral protease B expressed from the thermophilic *S. thermovulgaris* was estimated by measuring the enzyme’s activity against 5% each of azocasein, casein, and BSA at standard assay conditions. The enzyme exhibited the highest activity with casein, suggesting that casein is the best substrate of neutral protease B, while against BSA and azocasein, the enzyme activity was slightly decreased. The *K_m_* and *V_max_* values were 27.8 μM and 5824.2 μM/mL/min. 

#### 3.3.3. Effect of Metal Ions on Recombinant Protease Activity

The effect of metal ions on the recombinant neutral protease B was estimated in various metal ions, including Ca^2+^, Mg^+2^, Na^+2^, Cu^2+^, Zn^2+^, Ni^2+^, Mn^2+^, Hg^2+^, and Fe^2+^. The protease enzyme solution with 2 mM and 10 mM metal ions was incubated at 70 °C for 10 min. The relative protease activity is taken as the percentage of the enzyme activity compared to the control (without metal ions, i.e., 100.0 ± 2.30%). Mn^2+^, Fe^2+^, and Zn^2+^ at 10 mM significantly increase protease activity; Ca^2+^ at 10 mM did not affect enzyme activity. However, all other metal ions, including Zn^2+^ at 2 mM, decreased proteolytic activity (Table 2). 

#### 3.3.4. Effect of Organic Solvents on Recombinant Protease Activity

The effect of organic solvents on protease activity was assayed, and the results are shown in Table 3. The enzyme-neutral protease exhibited excellent stability in the presence of different organic solvents. The protease activity was slightly enhanced in the presence of benzene and chloroform (Table 3). 

#### 3.3.5. Effect of Surfactants on Recombinant Protease Activity

**Figure 6:**
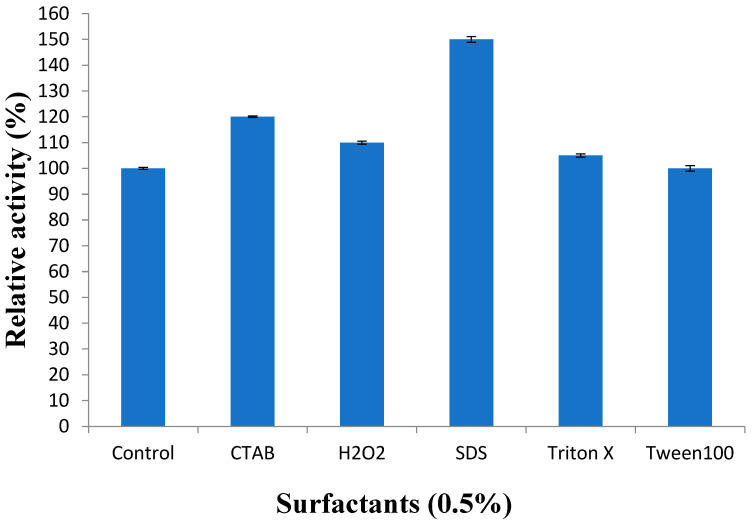
Effect of different surfactants and inhibitors on protease activity. Error bars represent the standard deviations of three measurements.

The protease was found stable against all studied surfactants, especially 0.5% SDS, with increased activity by 150% (Figure 6). 

Earlier reports have demonstrated that the proteases are stable in the presence of SDS and Triton X-100. The proteases show resistance in denaturation in the presence of SDS, urea, dithiothreitol, and guanidine hydrochloride. The alkaline proteases were found stable towards non-ionic surfactants such as Tween-20 (5%) and Triton X-100 (5%) and anionic surfactant (SDS 0.5% at 40 °C for one hour). The protease was found active in the presence of most of the surfactants. The neutral protease cloned and purified in this study has the highest stability in the presence of SDS, which is important for industrial use.

#### 3.3.6. Effect of Inhibitors on Recombinant Protease Activity

**Figure 7:**
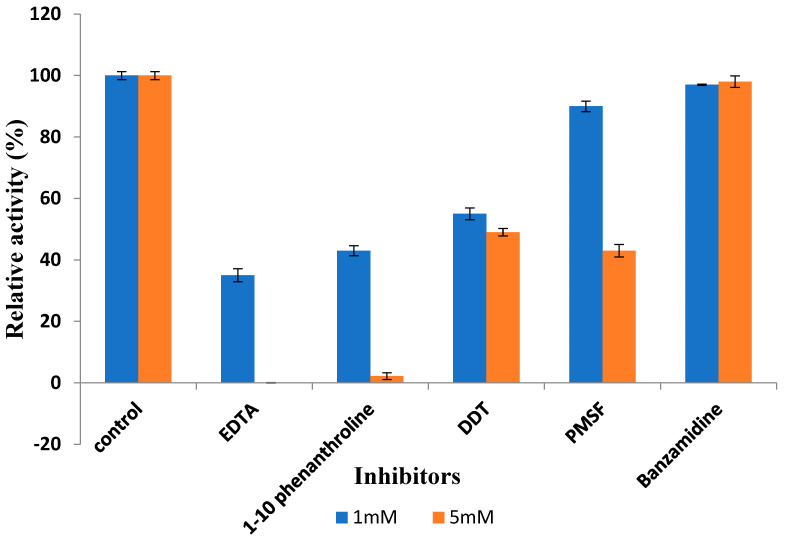
Effect of different inhibitors on protease activity at 1 mM and 5 mM concentrations. Error bars represent the standard deviations of three measurements.

The inhibitors of serine protease phenylmethylsulfonyl fluoride (PMSF) and benzamide did not affect the enzyme activity indicating that the enzyme did not belong to any of these catalytic groups (Figure 7). The enzyme activity was substantially reduced in the presence of 5 mM EDTA that is a divalent chelating agent and 87% of its activity was reduced by 5 mM 1–10 phenanthroline chelation of the Zn^2+^ ions and 43% by DTT.

## 4. Discussion

Microorganisms are well-known sources of enzymes [32,33]. Research is being performed on many microbial proteases due to their Commercial applications. One of the most essential aspects of industrial proteases is thermostability. Thermophilic microbes are the best source of thermostable enzymes [12]. An alkaline protease at an optimum temperature of 50 °C was produced from *Micrococcus* NH5 [8]. UV-90 mutant strain of *B. subtilis* produced protease at an optimum temperature of 50 °C [34]. The protease purified from the thermophilic *Bacillus* sp. strain SMIA-2 retained 70% of its activity after an incubation of 15 min at 70 °C, the enzyme [35]. Such enzymes are produced to achieve high efficiency and fast catalytic activity.

In this study, cloning, recombinant expression, purification, and characterization of neutral protease (NprB) were performed. Plasmid pET50-b (+) containing Nus tag was used as an expression vector to express protease (*nprB*) as a fusion protein. SDS-PAGE and Western blot analysis showed successful production of protease fused with the Nus tag and His tag; thus, the total size of the fusion protein is ~70 kDa. This is one of the strategies to increase the expression and solubility of produced protease. Investigations on cloning, expression, and characterization of different proteases showed varying molecular weights of enzymes falling within the range of 18–45 kDa [36,37]. 

Like previous studies, Metalloprotease (NprB) from *Streptomyces thermovulgaris* showed the highest activity at neutral pH. Proteases from the isolates of *Bacillus* sp. N-40 and *Bacillus* sp. SNR01 exhibited an optimum pH of 7 [38]. A protease from *Lactobacillus helveticus* also showed the highest activity at pH 7 [34]. The *Pseudomonas* produced an extracellular protease at an optimum pH of 7 [12]. However, some protease activity from *Bacillus licheniformis* LBLL, *Bacillus aquimaris* VITP4, and *Bacillus* sp. HS08 had an optimum pH of 8. Studies have reported a varying range of optimum temperatures for protease production. Protease enzyme purified from a hyperthermophilic *Bacillus* sp. strain HUTBS71 at 50 to 60 °C was stable for two hours, and approximately 84% of its activity was retained [39]. Hutadilok [40] reported that heat treatment of 5 min at 90 °C to the protease protein isolated from *Pseudomonas fluorescens* 07A was sufficient to inactivate the enzyme and the bacteria. The thermostable extracellular serine protease of *Aeribacillus pallidus* C10 retained more than 90% of its activity after two weeks of incubation at 40 °C in the presence of 40% of organic solvents such as methanol, ethanol, and isopropanol [15]. The extracellular protease produced by the *P. fluorescens* BJ–10 strain showed an optimum enzyme activity at 30 °C, and >94% of its activity was retained at 100 °C for 3 min of incubation, providing evidence of thermo-resistant characteristics [41,42]. In accordance with previous studies, protease proteins from various bacterial species, such as *B. pumilus* MCAS8 [43], *B. circulans* MTCC [6], and *B. laterosporus* AK1 [44] also exhibited the highest activities against the casein.

Metal ions are considered external factors that can affect enzymes catalytic stability and activity. Metal ions are well known to play an essential role as cofactors for enzyme activities, and often, they act as ion or salt bridges between the two adjacent amino acid residues. Cations are known for increasing the thermal stability of proteases and play a vital role in maintaining the active conformation of enzymes. Metal ions can protect the protease against thermal denaturation [4,15]. Our results are in accordance with previous studies. The protease has been found quite stable with higher activity in the presence of Zn^2+^, Ca^2+^, Fe^2+^ and Mn^2+^. Ions present in metalloprotease structure and are fundamental for the proteolytic activity [45]. Proteases are stable in the presence of most of the metal ions but exhibit an increased activity with Cu^2+^ and Fe^2+,^ while strong inhibition was found in the presence of Ni^2+^ and Co^2+^ [10]. A decrease in the activity was also observed in the presence of Ca^2+^, Hg^2+,^ and Co^2+^ [46]. Benzene and chloroform were reported to have adverse effects on the alkaline protease’s activity, while DMSO and toluene moderately decreased the enzyme activity. The ability of neutral proteases to remain stable without modifying them for enzyme stabilization is very important for various industrial applications. Protease stability in the presence of SDS makes this study more impactful, as in previous studies, only a few proteases are SDS stable [47,48]. A protease from *B. stearothermophilus* RM–67 was found to be inhibited by DFP, and the enzyme was classified as a serine protease [49]. In another study, the protease enzyme showed potent inhibition by the metal-chelating agent EDTA [50]. A protease enzyme was inhibited (i.e., 90%) with EDTA (10 mM) and 1,10-phenanthroline (10 mM), and therefore, the enzyme is classified as a metalloprotease [45]. 1 mM PMSF and TPCK inhibited the enzyme activity of a protease but not in the presence of 1,10-phenanthroline and EDTA [51]. PMSF inhibited the proteolytic activity of a protease, but in the presence of iodoacetate and 2-mercaptoethanol, 80 to 90% activity was retained [52]. 

## 5. Conclusions

The protease gene was successfully cloned and expressed in *E. coli* BL21. This is the first study in which the neutral protease enzyme from a thermophilic bacterium, *S. thermovulgaris*, was isolated and cloned for high expression. The enzyme was confirmed through SDS-PAGE and Western blotting, and after purification using Ni-NTA FPLC confirmed the eluted fractions contain protease of molecular mass of 20 kDa but fused with 55 KDa Nus tag; thus, the total size of the fusion protein is ~70 KDa., which is in accordance with the mass of other proteases. The enzyme showed a high 90-fold activity. The recombinant neutral protease B exhibited maximum activity at pH 7.0 but was stable in the pH range of 4–9. Protease NprB was identified as highly thermostable as it showed functional stability at a temperature range of 30–100 °C. The half-life of the enzyme at 70 °C was 4 h. The thermophilic recombinant neutral protease B (NprB) was strongly inhibited in the presence of EDTA (5 mM) and 1–10 phenanthroline (5 mM) but not affected by other inhibitors, which showed that the enzyme is a metalloprotease. Metals ions (i.e., Ca^2+^, Zn^2+^, Mn^2+^, Fe^2+^) were found to have an increasing effect on the protease activity, while Hg^2+^ had an inhibitory effect on the enzyme. The protease enzyme showed good activity towards casein, azocasein, and BSA, but the best substrate for this protease was casein, which had the highest activity. 

Due to its good properties, this protease from *S. thermovulgaris* can be a suitable candidate for use in various industries. 

## Figures and Tables

**Table 1:** Purification of recombinant neutral protease B (NprB).

Purification Steps	Total Protein (mg)	Total Activity (U)	Specific Activity (U/mg)	Purification Fold	Recovery Rate (%)
Crude extract	82	99,867.5	2155.6	-	100
Ammonium sulfate precipitation (80%)	65.3	90,875.4	2570.8	2.5	80.5
FPLC Ni-coated Hi-trap DEAE Sephadex	45.9	81,641.6	5698.5	15	90.5

**Table 2:** Effect of metal ions on protease activity.

Metal Ion	Relative Protease Activity (%)	
	Concentration (mM)	
	2 mM	10 mM
Cu^2+^	95.0 ± 0.1	90.0 ± 1.2
Na^+2^	90.4 ± 0.15	91 ± 0.8
Mg^2+^	90.1 ± 0.18	89.0 ± 0.09
Ni^2+^	58.5 ± 0.19	50.2 ± 1.7
Hg^2+^	78.9 ± 0.2	70.0 ± 0.3
Ca^2+^	85.2 ± 1.6	100 ± 0.01
Mn^2+^	170.2 ± 1.13	184.0 ± 0.2
Fe^2+^	190.6 ± 1.3	198.0 ± 1.6
Zn^2+^	75.5 ± 0.02	105.0 ± 0.11

**Table 3:** Effect of organic solvents on protease activity.

Organic Solvents	Concentration	Relative Protease Activity (%)
Control	0	100
Ethanol	10%	90.5 ± 0.9
Methanol	10%	95.4 ± 1.1
Hexane	10%	91.5 ± 0.8
Benzene	10%	105 ± 1.0
DMSO	10%	80 ± 0.9
Chloroform	10%	105 ± 1.1
Toluene	10%	88 ± 0.9

## Data Availability

All data presented in this article are available within the manuscript.
